# CNVs in neurodevelopmental disorders

**DOI:** 10.18632/oncotarget.4853

**Published:** 2015-07-13

**Authors:** Chun-Ting Lee, William J. Freed, Deborah C. Mash

**Affiliations:** Department of Neurology, Miller School of Medicine, University of Miami, Miami, Florida, USA

Copy number variations (CNVs) consist of duplications or deletions of chromosomal regions ranging from a few hundred to more than a million bases in size, and are likely to play a role in phenotypic diversity and evolution. Recent advances in the identification and mapping of CNVs among normal individuals and in model systems, using bioinformatics and hybridization-based methods, are beginning to shed light on the functional importance of CNVs.

Most CNVs are harmless; however, some are associated with human diseases including neurodevelopmental disorders. Hundreds of CNVs have been linked to neurological phenotypes, including autism, schizophrenia, and bipolar disorder. Some CNVs, such as duplications involving VIPR2 on 7q36.3 and deletions of NRXN1 on 2p16.3 have been identified in autism, schizophrenia, and bipolar disorder [1, 2]. Nevertheless, there are many cases in which CNVs are present only in specific diseases. For example, duplication of WNT3 and WNT9B at 17q21.31-17q21.32 and the 3p14.1 deletion that includes FOXP1 are reported in autism [3]. These observations suggest that CNVs associated with neurodevelopmental disorders have global effects on the transcriptome, affording new strategies for the treatment or prevention of neurodevelopmental disorders. However, uncovering associations between copy number changes and complex diseases requires novel model systems to elucidate the different mechanisms by which they influence neural development.

Recently, model systems for human genetic disorders have emerged based on the use of human pluripotent stem cells (hPSCs). These approaches include human embryonic stem cells (hESCs) and human induced pluripotent stem cells (hiPSCs). These models offer unique advantages for the study of developmental biology. hPSCs can be differentiated into various types of somatic cells including neurons to examine molecular and cellular mechanisms involved in disease causation. Differentiated hiPSCs have enabled the development of human in vitro model systems that capture the inherent pathologies based on the genetic background of the source material. These stem cell lines derived from patients with various diseases offer unique advantages for the study of inherited neurological disorders. Such patient-specific hiPSCs allow examination of underlying genetic factors in human organ systems that affect neural development.

MRI scans and post-mortem studies of autism spectrum disorders suggest abnormal brain development due to initial brain overgrowth followed by premature growth arrest [4]. Duplications of CNVs at the 17q21.31 and 17q21.32 regions have been reported in 25 genetic studies of autism (CNV catalog of SFARI Gene database). Of particular interest is the duplication mapping of chromosome 17q21.31-17q21.32 covering WNT3 and WNT9B identified in autism [3]. Lee and coworkers have taken advantage of laboratory- and cell line-specific genomic variations in hPSCs and reported that hPSC lines carrying the WNT3 and WNT9B CNV exhibit enhanced neural differentiation [5]. In hPSC lines with amplified WNT3/WNT9B, WNT9B signaling, the non-canonical Rho/JNK pathway leads to the loss of pluripotency. WNT3 through canonical/β-catenin signaling accelerated subsequent neuronal specification in this model system. The addition of growth factor bFGF switches the role of WNT9B/JNK to WNT3/β-catenin as the dominant regulator to induce neuronal differentiation. These findings connect a biological function to a specific CNV and implicate WNT signaling as a possible biological driver of pathogenesis of autism. This work demonstrates that the hPSC-based neuronal model may provide a platform for medication development for autism, including therapeutic strategies that target WNT pathways.

Schizophrenia is a complex genetic neurodevelopmental disorder that carries increased CNV burden. Using iPSC technology, Brennand and coworkers showed that hiPSC neurons from schizophrenia patients exhibit diminished neuronal connectivity and reduced neurite outgrowth and glutamate receptor expression [6]. In addition, three CNV-related genes, CSMD1, MYH1, and MYH4 show altered neuronal expression with genotype. However, the relevant cellular functions and phenotypic consequences of these schizophrenia CNVrelated genes remain unidentified.

Novel genome engineering approaches using ZFNs, TALENs, and CRISPRs have been applied to study specific functions in hPSCs. These tools provide the opportunity for correction of disease-causing mutations in patient-derived hiPSC models. Importantly, genetic and epigenetic instabilities, including enhanced de novo CNV induction have been observed during long-term propagation of hPSCs [7]. These confounding factors might contribute to the pathophenotypes observed in the patient-derived hiPSCs. Caution should be taken in order to avoid these confounding factors. It is necessary to incorporate disease-associated genetic alterations into the genomes of wild type hPSCs and compare to the same parental hPSC line in addition to the use of genome editing to correct the disease mutations in patient-derived hiPSCs. This approach will allow scientists to establish causation of genetic-inherited disease by comparing genotype to phenotype directly.

Stem cell technology using patient-derived hiPSCs with well-defined CNVs recapitulates the developmental programs that guide formation or malformation of brain region-specific neurons. The application of genomeediting tools to studies of hiPSCs will give important insights into the genome structural changes that underlie fundamental disease processes at the cellular level. These combined approaches are a boost to developmental neuroscience that could be transformative in the years ahead.
